# Impact of Squamous and Glandular Differentiation on Oncologic Outcomes in Upper and Lower Tract Urothelial Carcinoma

**DOI:** 10.1371/journal.pone.0107027

**Published:** 2014-09-05

**Authors:** Young Ju Lee, Kyung Chul Moon, Chang Wook Jeong, Cheol Kwak, Hyeon Hoe Kim, Ja Hyeon Ku

**Affiliations:** 1 Department of Urology, Seoul National University College of Medicine, Seoul, Korea; 2 Department of Pathology, Seoul National University College of Medicine, Seoul, Korea; Istituto dei tumori Fondazione Pascale, Italy

## Abstract

**Purpose:**

To investigate the prognostic significance of squamous and/or glandular differentiation in urothelial carcinoma (UC).

**Materials and Methods:**

Among 800 consecutive patients who underwent radical cystectomy or nephroureterectomy at our institution from January 1990 to December 2010, 696 patients were included for the analysis. Clinicopathologic variables were compared according to the presence of squamous and/or glandular differentiation and the tumor location.

**Results:**

A total of 51 (7.3%) patients had squamous and/or glandular differentiation. Patients with squamous and/or glandular differentiation had higher pathological T stage (p<0.001) and grade (p<0.001) than those with pure form of UC. After the median follow-up of 55.2 months, 84 (24.6%) and 82 (23.1%) died of upper urinary tract UC and UC of bladder, respectively. Patients with squamous and/or glandular differentiation in upper urinary tract UC showed poorer cancer-specific survival (CSS) (p<0.001) and overall survival (OS) (p<0.001) than those with pure form in upper urinary tract UC (p<0.001), but not in UC of bladder (p = 0.178 for CSS and p = 0.172 for OS). On multivariate Cox regression analysis, squamous and/or glandular differentiation was an independent predictor of CSS (hazard ratio [HR] 1.76; 95% confidence interval [CI] 1.08–2.85, p = 0.023), but it was not associated with OS (HR 1.52; 95% CI 1.00–2.32, p = 0.051).

**Conclusions:**

The presence of variant histology could be associated with poorer survival outcome in patients with UC. Squamous and/or glandular differentiation is associated with features of biologically aggressive disease and an independent predictor of CSS.

## Introduction

Bladder cancer is the 4th leading cause of new cancer cases and 8th leading cause of cancer-related mortality in males in the United States [1]. Bladder cancer accounted for 73,510 new cases of cancer and 14,880 cancer-related deaths in the United States during 2012 [1]. Bladder tumors are the most common malignancy of the urinary tract, while upper urinary tract carcinomas are relatively uncommon comprising 5–10% of all urinary tract carcinomas [2], [3]. In cancers involving the urinary tract, the most common histology is pure urothelial carcinoma (UC). In the United States, 90% to 95% of bladder cancers are pure UC and the remaining consists of UC with histological variants or non-UC. Squamous differentiation is the most common histological variant of UC, constituting nearly 10% of bladder tumor, followed by glandular differentiation [4]–[7].

Pure non-UC are usually diagnosed and treated at an advanced stage and higher grade, and are associated with more aggressive behavior and worse survival when compared to pure UC [8]. However, it is unclear whether this result can be applied to UC with histological variants although variant forms of UC also correspond to high grade diseases and advanced stages [4]–[6], [9]–[13].

Not much research has been performed regarding the impact of squamous and/or glandular histologic variants on oncologic outcomes in urothelial cancer [4], [11]. Because little evidence exists in the literature about the prognostic significance of histological variants in UC, we investigated the prognostic significance of squamous and/or glandular differentiation in UC.

## Materials and Methods

### Patient characteristics

After obtaining the approval of the institutional review board, a retrospective medical chart review was performed for the records of 800 consecutive patients diagnosed with urinary tract carcinoma after radical cystectomy or nephroureterectomy at our institution between January 1990 and December 2010. Patients with incomplete data (n = 3), non-urothelial cancer (n = 19) or urothelial cancers of other variant histology (n = 21), metastatic disease at diagnosis (n = 16) and the history of neoadjuvant chemotherapy (n = 45) were excluded, leaving 696 patients for the analysis. A total of 341 upper urinary tract UC and 355 UC of the bladder were included for the analysis including 27 and 24 squamous and/or glandular variants, respectively. The work-up, surgery, pathologic review, and follow-up have been described previously in detail [14], [15].

### Surgical Procedure and Pathologic evaluation

Radical cystectomy with bilateral pelvic lymph node dissection or radical nephroureterectomy was performed by various surgeons at our institution using standard techniques. For the patients who underwent radical cystectomy, the extent of lymph node dissection was according to the discretion of individual surgeons. For patients who underwent radical nephroureterectomy, lymph node dissection was performed if there was an enlarged lymph node on preoperative computed tomography (CT) scan. Tumor grade was assigned according to the 1973 World Health Organization (WHO) grading system [16]. Pathologic stage was determined according to the 2002 WHO Tumor-node-metastasis (TNM) classification of 6th American Joint Committee on Cancer (AJCC) [17]. The subtypes of UC were defined according to the 2004 WHO publication [18].

### Follow-up

Follow-up was done according to the institutional protocol. In general, patients were followed up at every 3–4 months during the first year, semiannually for the second year, and annually thereafter. Follow-up examinations consisted of physical examination, lab tests including urine cytology, chest X-rays, and renal ultrasound. The CT scan of the abdomen and pelvis was carried out annually. Clinical outcomes were estimated from the date of the surgery to the date of death or last follow-up. For deceased patients during the follow-up, the causes of death were determined by the treating physician with reference to the chart review corroborated by death certificates.

### Statistical analysis

Continuous variables according to the presence of the squamous and/or glandular differentiation were compared with Student’s t-test and categorical variables were compared with chi-square test. Cancer-specific survival (CSS) and overall survival (OS) stratified by the presence of squamous and/or glandular differentiation were estimated using Kaplan-Meier method, and differences between the two groups were compared by log-rank test. To estimate the predictive factors of CSS and OS after the surgery, univariate and multivariate Cox proportional hazard analysis were performed. All tests were 2-sided with p<0.05 considered to be significant. Statistical analysis was performed using IBM SPSS Software, version 21 (SPSS, Chicago, Illinois, USA).

### Ethics Statement

This research was approved by the institutional review board of Seoul National University Hospital and conducted following the principles as expressed in the Declaration of Helsinki. Written informed consent was exempted and approved by the institutional review board because this retrospective research did not affect the clinical course of any patient. Patient records were anonymized and de-identified prior to the analysis.

## Results

Table 1 lists the clinicopathologic demographics of patients stratified with the location of tumor and the presence of histological variation. Median age at surgery was 63.2 and 62.9 years for patients with pure UC and UC with squamous and/or glandular differentiation, respectively. Among 696, 51 (7.3%) had UC with squamous and/or glandular differentiation. In 341 patients who underwent radical nephroureterectomy, 23 (6.7%) had squamous and 4 (1.2%) had glandular differentiation, whereas 21 (5.9%) had squamous and 3 (0.8%) had glandular differentiation in 355 patients who underwent radical cystectomy.

**Table 1:** Patient characteristics.

	Total			Upper urinary tract cancer			Bladder cancer		
	Pure form	Variantform	P value	Pureform	Variantform	P value	Pureform	Variantform	P value
No. of patients	645	51		314	27		331	24	
Age, years			0.331			0.251			0.822
Mean	62.7	61.3		63.1	60.7		62.3	61.8	
IQR	56.8–70.0	54.3–69.4		56.4–70.5	54.3–68.9		57.4–69.4	54.2–70.6	
Sex			0.958			0.147			0.505
Male	542 (84.0%)	43 (84.3%)		275 (87.6%)	26 (96.3%)		295 (89.1%)	21 (87.5%)	
Female	103 (16.0%)	8 (15.7%)		39 (12.4%)	1 (3.7%)		36 (10.9%)	3 (12.5%)	
Pathological T category			<0.001			<0.001			0.024
pT0	37 (5.7%)	0 (0.0%)		0 (0.0%)	0 (0.0%)		37 (11.2%)	0 (0.0%)	
pTis	33 (5.1%)	0 (0.0%)		1 (0.3%)	0 (0.0%)		32 (9.7%)	0 (0.0%)	
pTa	69 (10.7%)	1 (2.0%)		53 (16.9%)	0 (0.0%)		16 (4.8%)	1 (4.2%)	
pT1	143 (22.2%)	1 (2.0%)		81 (25.8%)	0 (0.0%)		62 (18.7%)	1 (4.2%)	
pT2	120 (18.6%)	9 (17.6%)		55 (17.5%)	3 (11.1%)		65 (19.6%)	6 (25.0%)	
pT3	207 (32.1%)	36 (70.6%)		121 (38.5%)	23 (85.2%)		86 (26.0%)	13 (54.2%)	
pT4	36 (5.6%)	4 (7.8%)		3 (1.0%)	1 (3.7%)		33 (10.3%)	3 (12.5%)	
Tumor grade			<0.001			<0.001			0.003
0	37 (5.7%)	0 (0.0%)		0 (0.0%)	0 (0.0%)		37 (11.2%)	0 (0.0%)	
1	43 (6.7%)	0 (0.0%)		39 (12.4%)	0 (0.0%)		4 (1.2%)	0 (0.0%)	
2	303 (47.0%)	5 (9.8%)		202 (64.3%)	4 (14.8%)		101 (30.5%)	1 (4.2%)	
3	262 (40.6%)	46 (90.2%)		73 (23.2%)	23 (85.2%)		189 (57.1%)	23 (95.8%)	
LVI			0.034			0.007			0.580
Absent	480 (74.4%)	31 (60.8%)		255 (81.2%)	16 (59.3%)		225 (68.0%)	15 (62.5%)	
Present	165 (25.6%)	20 (39.2%)		59 (18.8%)	11 (40.7%)		106 (32.0%)	9 (37.5%)	
Associated CIS			0.120			0.432			0.024
Absent	526 (81.6%)	46 (90.2%)		287 (91.4%)	24 (88.9%)		239 (72.2%)	22 (91.7%)	
Present	119 (18.4%)	5 (9.8%)		27 (8.6%)	3 (11.1%)		92 (27.8%)	2 (8.3%)	
Positive surgical margin			0.005			0.023			0.178
Absent	615 (95.3%)	44 (86.3%)		303 (96.5%)	23 (85.2%)		312 (94.3%)	21 (87.5%)	
Present	30 (4.7%)	7 (13.7%)		11 (3.5%)	4 (14.8%)		19 (5.7%)	3 (12.5%)	
Pathological N category			0.151			0.035			0.299
pN−	301 (46.7%)	20 (39.2%)		37 (11.8%)	3 (11.1%)		264 (79.8%)	17 (70.8%)	
pN×	265 (41.1%)	20 (39.2%)		265 (84.4%)	20 (74.1%)		0 (0.0%)	0 (0.0%)	
pN1–3	79 (12.2%)	11 (21.6%)		12 (3.8%)	4 (14.8%)		67 (20.2%)	7 (29.2%)	
ACH			<0.001			<0.001			0.032
Not done	492 (76.3%)	22 (43.1%)		246 (78.3%)	9 (33.3%)		246 (74.3%)	13 (54.2%)	
Done	153 (23.7%)	29 (56.9%)		68 (21.7%)	18 (66.7%)		85 (25.7%)	11 (45.8%)	

Abbreviations: IQR = interquartile range, BMI = body mass index, ASA = American Society of Anesthesiologists, LVI = Lymphovascular invasion, CIS = carcinoma *in* *situ,* NACH = neoadjuvant chemotherapy, ACH = adjuvant chemotherapy.

Age and gender were not different according to the presence of the squamous and/or glandular differentiation. Pathologic T3/T4 tumors were more common in UC of squamous and/or glandular variants than pure UC (p<0.001). Lymphovascular invasion was more common in patients of histological variants in upper urinary tract (p = 0.007), whereas the prevalence of it was not different among patients with UC of the bladder according to the histological variation. The proportion of patients receiving an adjuvant chemotherapy was higher in patients having the squamous and/or glandular differentiation (p<0.001).

Median follow-up duration was 55.2 months (interquartile range 30.0–95.3). In upper urinary tract UC, median follow-up duration was 66.8 and 46.0 months for pure form and variant form, respectively. Among 84 patients who died of upper urinary tract UC, 14 (16.7%) had variant form. In patients with UC of the bladder, median follow-up duration was 42.4 and 37.2 months for pure form and variant form, respectively. Among 82 patients who died of UC of the bladder, 8 (9.8%) had variant form histology. As an all-cause mortality, 128 (37.5%) deaths occurred in patients with upper urinary tract UC and 120 (33.8%) had died in those with UC of the bladder.

**Figure 1:**
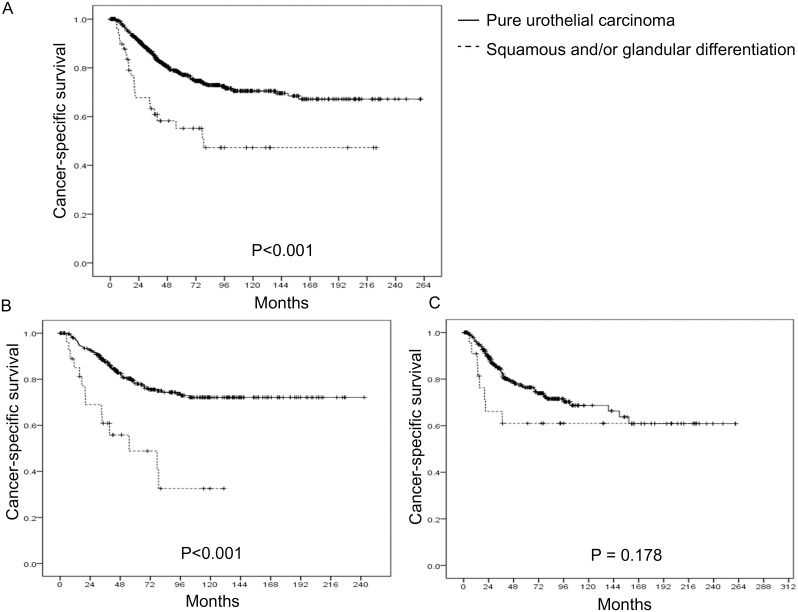
Kaplan-Meier curves for cancer-specific survival stratified by the presence of histological variant. (A) Total (p<0.001). (B) Upper urinary tract urothelial carcinoma (p<0.001). (C) Urothelial carcinoma of bladder (p = 0.178).

**Figure 2:**
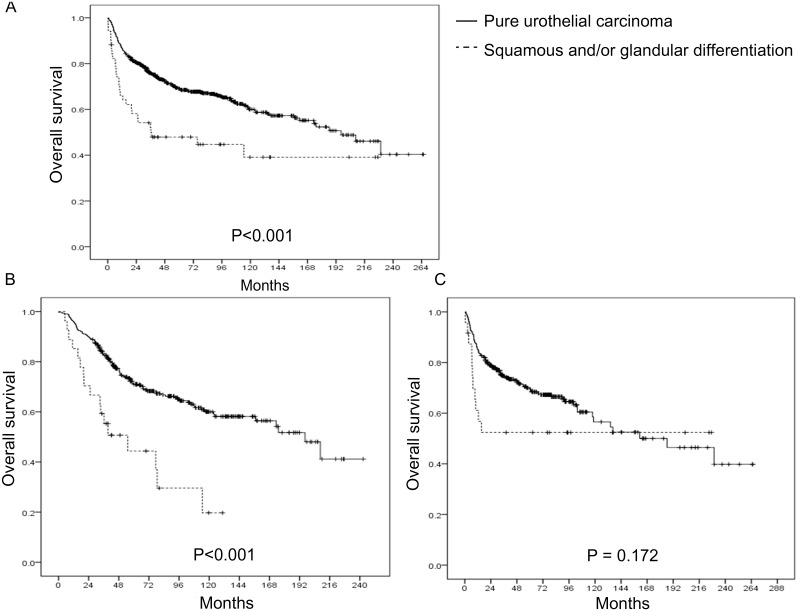
Kaplan-Meier curves for overall survival stratified by the presence of histological variant. (A) Total (p<0.001). (B) Upper urinary tract urothelial carcinoma (p<0.001). (C) Urothelial carcinoma of bladder (p = 0.172).

Figure 1 and 2 show Kaplan-Meier curve for CSS and OS. Patients with squamous and/or glandular differentiation showed worse survival than those with pure UC. Kaplan-Meier curves for CSS stratified by the histologic type showed that patients with squamous and/or glandular histology had worse CSS when compared to those with pure UC (p<0.001) (Figure 1A). Subgroup analysis was performed according to the location of the tumor. In upper urinary tract, Kaplan-Meier curve showed that the presence of histological variant was associated with worse CSS (p<0.001) (Figure 1B). When it was confined to the bladder, CSS was not different according to the presence of histological variant (p = 0.178) (Figure 1C).

Kaplan-Meier curves for OS stratified by the presence of histological variant showed that patients with squamous and/or glandular histology had worse OS when compared to those with pure UC (p<0.001) (Figure 2A). The presence of histological variant was associated with significantly worse OS when the tumors were located in upper urinary tract (p<0.001) (Figure 2B). However, the OS of the bladder cancers were not affected by the presence of histological variants (p = 0.172) (Figure 2C).

The results of Cox proportional hazard regression analysis for CSS and OS are shown in Table 2 and 3. Multivariate Cox proportional hazard regression analysis revealed that older age (p = 0.001; hazard ratio [HR] 1.03), higher pT stages (pT2 vs. ≤pT1; p = 0.006; HR 2.13 and ≥pT3 vs. ≤pT1; p<0.001; HR 3.71), the presence of lymphovascular invasion (present vs. absent; p = 0.001; HR 1.79), positive surgical margin (positive vs. negative; p = 0.031; HR 1.80), nodal metastasis (pN+ vs. pN−; p = 0.004; HR 1.94) and the presence of histological variants (present vs. absent; p = 0.025; HR 1.74) were significant independent predictors of CSS (Table 2).

**Table 2:** Univariate and multivariate Cox proportional hazard regression analysis of cancer-specific survival.

	Univariate		Multivariate	
	HR (95% CI)	P value	HR (95% CI)	P value
Age, year	1.02 (1.00–1.04)	0.015	1.03 (1.01–1.05)	0.001
Sex				
Male	Reference		Reference	
Female	0.99 (0.66–1.48)	0.946	0.94 (0.62–1.43)	0.787
Pathological T category				
≤pT1	Reference		Reference	
pT2	2.72 (1.63–4.54)	<0.001	2.13 (1.25–3.63)	0.006
≥pT3	5.42 (3.55–8.27)	<0.001	3.71 (2.23–6.15)	<0.001
Tumor grade				
≤II	Reference		Reference	
III	1.79 (1.32–2.43)	<0.001	0.97 (0.68–1.39)	0.867
Lymphovascular invasion				
Absent	Reference		Reference	
Present	2.94 (2.16–3.98)	<0.001	1.79 (1.27–2.52)	0.001
Associated carcinoma *in* *situ*				
Absent	Reference		Reference	
Present	0.93 (0.61–1.41)	0.722	0.92 (0.59–1.44)	0.716
Positive surgical margin				
Negative	Reference		Reference	
Positive	2.94 (1.80–4.79)	<0.001	1.80 (1.06–3.06)	0.031
Pathological N category				
pN−	Reference		Reference	
pN×	1.09 (0.77–1.54)	0.639	0.93 (0.52–1.67)	0.808
pN+	3.45 (2.31–5.13)	<0.001	1.94 (1.24–3.05)	0.004
Adjuvant chemotherapy				
Not done	Reference		Reference	
Done	2.47 (1.82–3.35)	<0.001	0.99 (0.66–1.48)	0.968
Tumor location				
Bladder	Reference		Reference	
Upper urinary tract	0.89 (0.66–1.20)	0.444	0.87 (0.51–1.48)	0.600
Variant form				
Absent	Reference		Reference	
Present	2.47 (1.58–3.86)	<0.001	1.74 (1.07–2.83)	0.025

Abbreviations: HR = hazard ratio, CI = confidence interval.

**Table 3:** Univariate and multivariate Cox proportional hazard regression analysis of overall survival.

	Univariate		Multivariate	
	HR (95% CI)	P value	HR (95% CI)	P value
Age, year	1.04 (1.03–1.06)	<0.001	1.05 (1.03–1.06)	<0.001
Sex				
Male	Reference		Reference	
Female	0.99 (0.71–1.38)	0.937	0.92 (0.65–1.31)	0.655
Pathological T category				
≤pT1	Reference		Reference	
pT2	1.88 (1.25–2.83)	0.003	1.60(1.05–2.46)	0.030
≥pT3	4.07 (2.97–5.59)	<0.001	3.48 (2.38–5.09)	<0.001
Tumor grade				
≤II	Reference		Reference	
III	1.65 (1.29–2.12)	<0.001	0.96 (0.72–1.28)	0.780
Lymphovascular invasion				
Absent	Reference		Reference	
Present	2.33 (1.80–3.00)	<0.001	1.57 (1.18–2.10)	0.002
Associated carcinoma *in* *situ*				
Absent	Reference		Reference	
Present	0.93 (0.66–1.32)	0.687	1.00 (0.69–1.46)	0.989
Positive surgical margin				
Negative	Reference		Reference	
Positive	2.81 (1.85–4.25)	<0.001	1.86 (1.19–2.91)	0.007
Pathological N category				
pN−	Reference		Reference	
pN×	1.06 (0.80–1.40)	0.698	0.93 (0.57–1.50)	0.755
pN+	2.76 (1.96–3.89)	<0.001	1.74 (1.19–2.56)	0.005
Adjuvant chemotherapy				
Not done	Reference		Reference	
Done	1.81 (1.40–2.35)	<0.001	0.83 (0.60–1.17)	0.286
Tumor location				
Bladder			Reference	
Upper urinary tract	0.91 (0.71–1.16)	0.438	0.96 (0.61–1.50)	0.842
Variant form				
Absent	Reference		Reference	
Present	2.01 (1.36–2.98)	0.001	1.52 (1.00–2.32)	0.053

Abbreviations: HR = hazard ratio, CI = confidence interval.

Using the same variables, age (p<0.001; HR, 1.05), pT stages (pT2 vs. ≤pT1; p = 0.030; HR 1.60 and ≥pT3 vs. ≤pT1; p<0.001; HR 3.48), lymphovascular invasion (present vs. absent; p = 0.002; HR 1.57), surgical margin status (positive vs. negative; p = 0.007; HR 1.86) and pN stages (pN+ vs. pN−; p = 0.005; HR 1.74) were significant independent prognostic factors of OS on multivariate Cox proportional hazard analysis (Table 3). However, the presence of histological variants was not associated with differences in OS (p = 0.053).

## Discussion

UC with squamous and/or glandular differentiation is the most common type of histological variants in UC but the prognosis of it is unclear [19]. In fact, the outcomes of pure squamous cell carcinoma have not been well defined, too. According to the SEER database, squamous cell carcinoma of the bladder appears to be more aggressive than UC of the bladder after adjusting for stage and other prognostic factors, except for the cases whose tumors were confined to the bladder wall or treated with an initial cystectomy [20]. Ploeg et al. [21] analyzed the nationwide data of Netherlands Cancer Registry and found that patients with muscle invasive squamous cell carcinoma had worse survival regardless of their stage. On the contrary, Nishiyama et al. [22] studied 1,311 Japanese patients who underwent radical cystectomy which included 89 patients of non-urothelial carcinoma and found that the histological subtype was not an independent predictor of OS. A recent multicenter study showed that cancer-specific progression and mortality of squamous cell carcinoma were not different significantly from patients with UC even after adjustment for stages [4]. Honma et al. [23] reported that a concomitant squamous cell carcinoma component in the specimen was an independent predictor of local recurrence after radical cystectomy. Ehdaie et al. [10] reported that CSS or OS of patients with squamous differentiation were worse than those with squamous cell carcinoma of the bladder at a median follow-up of 44 months. In another study, squamous differentiation was an adverse independent predictor of CSS after radical cystectomy [24].

Generally, UC with squamous and/or glandular differentiation is associated with higher stage and grade at presentation than pure UC [25]. In bladder cancer after radical cystectomy, the impact of squamous and/or glandular differentiation on survival was inconsistent between the literatures. Kim et al. [5] reported that patients with squamous and/or glandular differentiation were more likely to have extravesical tumors and node-positive diseases, after the retrospective review of 1,013 patients who underwent radical cystectomy. However, it was not associated with worse survival at a median follow-up of 11.4 years in their study. Xylinas et al. [9] reported that histological variants were associated with significantly higher risk of recurrence and worse cancer-specific mortality in univariate analysis. However, it was not an independent predictor of cancer-specific mortality in multivariate analysis when adjusted with age, gender, pathologic stage, pathologic grade, nodal metastasis, the presence of concomitant carcinoma *in* *situ*, lymphovascular invasion, and positive surgical margin. Our result revealed that the presence of squamous and/or glandular differentiation is associated with worse CSS in multivariate Cox regression analysis.

In upper urinary tract urothelial cancer, few reports exist regarding the impact of variant histology on oncologic outcome. Rink et al. [11] reported that patients with variant histology tended to have more disease recurrence and cancer-specific mortality than those with pure upper urinary tract UC. However, it was not an independent predictor of disease recurrence or cancer-specific mortality in multivariate Cox regression analysis. Regarding the impact of histologic variants according to the tumor location, Rink et al [12] also reported that tumor location was not associated with CSS, but in pT4 tumors, patients with ureteral or pelvocalyceal tumors were more likely to experience disease recurrence and mortality compared to those having tumors in the bladder after the radical surgery. In our result, although Kaplan-Meier curve showed worse survival outcomes of histological variants in upper urinary tract UC compared to pure UC, the tumor location was not associated with either OS or CSS in Cox regression analysis.

Due to the high probability of relapse, some authors advocate neoadjuvant chemotherapy for patients showing UC with histological variants. A recent retrospective review of the SWOG study showed that the presence of squamous or glandular differentiation in locally advanced bladder cancer did not indicate resistance to MVAC therapy [26]. Rather this could warrant neoadjuvant chemotherapy. In contrast, other studies revealed that squamous cell carcinoma and UC with squamous differentiation were less chemosensitive than pure forms of UC, thus predicting poor response to chemotherapy [27]. Further studies with larger numbers of patients with squamous and/or glandular differentiation of UC are still needed to evaluate the role of neoadjuvant chemotherapy.

There are a few limitations of the present study. It is retrospective in nature and is subject to have inherent biases in a patient selection and treatment choice. It is a single institutional data and includes relatively small number of patients. However, this limitation is unavoidable considering the scarcity of the histological variants of UC. Further studies including larger numbers of patients are still needed to evaluate the role of variant forms. In addition, it was not possible to provide the percentage of morphological differentiation in the whole specimens because this was a retrospective review and encompasses the data of almost two decades.

In conclusion, squamous and/or glandular differentiation is associated with an advanced stage and higher grade, when compared to pure UC. In the present study, the presence of squamous and/or glandular differentiation was an important independent prognostic factor for CSS. Particularly, squamous and/or glandular differentiation of UC in upper urinary tract was associated with poorer outcomes of CSS and OS.
